# *Fucus vesiculosus*-Derived Fucoidan Mitigates Recognition Memory Deficits in Association with Modulation of the Microbiota–Gut–Brain Axis in Adenine-Induced Chronic Kidney Disease Mice

**DOI:** 10.3390/md24090323

**Published:** 2026-09-16

**Authors:** Xiaobo Kang, Zhiyou Yang, Zhihui Ma, Jinfeng Hong, Yi Zhang, Longjian Zhou

**Affiliations:** 1 College of Food Science and Technology, Shenzhen Institute of Guangdong Ocean University, Guangdong Provincial Key Laboratory of Aquatic Product Processing and Safety, Guangdong Province Engineering Laboratory for Marine Biological Products, Zhanjiang Municipal Key Laboratory of Marine Drugs and Nutrition for Brain Health, Guangdong Ocean University, Zhanjiang 524088, China; k17701892041@126.com (X.K.); zyyang@gdou.edu.cn (Z.Y.); mazhihui@stu.gdou.edu.cn (Z.M.); jinfeng_hong@163.com (J.H.); zhangyi@gdou.edu.cn (Y.Z.); 2 Collaborative Innovation Center of Seafood Deep Processing, Dalian Polytechnic University, Dalian 116034, China

**Keywords:** fucoidan, chronic kidney disease, recognition memory, gut microbiota, serum metabolomics

## Abstract

Chronic kidney disease (CKD) induces systemic oxidative stress and inflammation, leading to secondary cognitive impairment. The mechanisms by which marine-derived fucoidan exerts neuroprotection remain incompletely understood. Here we show that oral administration of *Fucus vesiculosus*-derived fucoidan improved object-recognition and object-location preferential indices, attenuated renal tubulointerstitial fibrosis, and reduced systemic oxidative and inflammatory markers in adenine-induced CKD mice. In hippocampal tissue, fucoidan attenuated oxidative stress and neuroinflammation, as reflected by decreased MDA and ROS levels, improved SOD and CAT activities, reduced TNF-α and IL-1β levels, and increased IL-10 and IL-4 levels. Integrating 16S rRNA sequencing with non-targeted serum metabolomics, we show that fucoidan is associated with modulation of CKD-induced gut dysbiosis, a lower relative abundance of circulating, putatively annotated uremic-toxin features, and altered lipid and amino acid metabolic pathways. Multi-omics correlation analysis identified significant associations among specific bacterial taxa, aberrant serum metabolites, and host physiological parameters. These results indicate that the renoprotective and neuroprotective effects of fucoidan are associated with gut microbiota regulation and altered circulating uremic-toxin features, highlighting its potential as a marine-derived prebiotic for CKD-associated complications.

## 1. Introduction

CKD affects approximately 10–13% of the global population and is becoming increasingly considered as a systemic disorder rather than a renal disease [1,2]. A substantial extra-renal consequence is CKD-related cognitive impairment, which is common in advanced stages and is consistently linked to poorer survival and reduced quality of life [3,4]. The accumulation of uremic toxins in the body causes neuroinflammation and is the main cause of central nervous system (CNS) damage [5,6]. At present, pharmacological management of CKD is largely directed at slowing disease progression and managing cardiovascular and disease-related complications. Guideline-based care includes blood-pressure control with renin–angiotensin system (RAS) blockade using angiotensin-converting enzyme (ACE) inhibitors or angiotensin II receptor blockers (ARBs), and sodium–glucose cotransporter 2 (SGLT2) inhibitors in eligible patients; additional therapies are used to manage specific CKD complications, including anaemia and CKD-mineral and bone disorders [7]. However, these kidney- and complication-directed therapies have limited and largely indirect effects on central nervous system (CNS) symptoms [8,9]. Although improved blood-pressure control, correction of anaemia, and reduction in circulating uremic toxins may partially attenuate cognitive decline, no established intervention specifically targets CKD-associated cognitive impairment, which therefore remains a major unmet clinical need. Consequently, the identification of novel interventions that simultaneously preserve kidney function and mitigate the risk of subsequent cognitive impairment has become a clinical priority [10,11].

The pathophysiological mechanisms of cognitive impairment induced by CKD are complexly associated with the gut-kidney-brain axis [12,13]. With progressive renal deterioration, metabolic wastes accumulate systemically, further aggravating intestinal barrier impairment and gut microbiota dysbiosis [14,15]. Such microbial dysbiosis is an opportunity for expansion of proteolytic bacteria that can ferment dietary aromatic amino acids into uremic precursors such as indole and p-cresol [14]. After hepatic metabolism, these precursors generate circulating uremic neurotoxins, such as indoxyl sulfate and p-cresyl sulphate [16,17]. These accumulated toxins, after breaching the impaired blood–brain barrier, trigger oxidative stress and neuroinflammation in the hippocampal circuits, resulting in cognitive deficits [18]. Thus, targeting the gut microbiome appears to be a viable therapeutic strategy to attenuate both CKD development and its associated neurological consequences.

Marine-derived sulfated polysaccharides such as fucoidan have been well-documented to possess anti-inflammatory, antioxidant and gut modulating properties [19,20,21]. Previously, we reported that fucoidan from *Laminaria japonica* attenuated CKD-associated cognitive decline and decreased oxidative stress in the brain, an effect mediated via the modulation of microglial polarization and the GSK3β/Nrf2 signaling pathway in the brain [22]. That work, however, focused on localized neuroprotection, and the mechanisms by which an essentially unabsorbed macromolecular polysaccharide can nonetheless be linked to protection of remote organs remain unresolved. Fucoidan is a macromolecule of high molecular weight and high polarity with poor oral bioavailability [23,24], direct penetration to the brain parenchyma is unlikely. Following ingestion, fucoidan is largely resistant to the acidic gastric environment and to hydrolysis by mammalian digestive enzymes, so that the intact macromolecule reaches the colon largely undegraded. Only a minor fraction of low-molecular-weight fragments (oligosaccharides and partially desulfated derivatives) is absorbed across the intestinal epithelium, accounting for its limited systemic bioavailability [23,24,25]. In the colon, fucoidan may be utilized and catabolized by resident microbiota into smaller fermentable products, potentially contributing to short-chain fatty acid (SCFA) production and enrichment of polysaccharide-degrading taxa [26,27,28]. This gut-centric digestive and catabolic fate underpins our hypothesis that the primary interactions of fucoidan occur in the gastrointestinal tract and that the central neuroprotective effects are indirect. Unlike our previous study, which focused on the GSK3β/Nrf2 pathway in the brain, the current work provides a more comprehensive view by integrating microbiome and metabolomic data and highlights the gut microbiota as a potential key interface through which fucoidan exerts its effects.

In addition, as structural features, particularly sulfate content, are recognized determinants of the bioactivity of fucoidans [20,26], the fucoidan used in the present study was a highly sulfated fucoidan extracted from *Fucus vesiculosus*. This study was to investigate whether oral administration of fucoidan could alleviate the CKD-induced CNS damage in association with modulation of the gut microbiota and altered serum metabolite profiles, by using 16S rRNA gene sequencing in combination with untargeted serum metabolomics.

## 2. Results

### 2.1. Structural Characterization of Fucoidan from Fucus Vesiculosus

**Figure 1:**
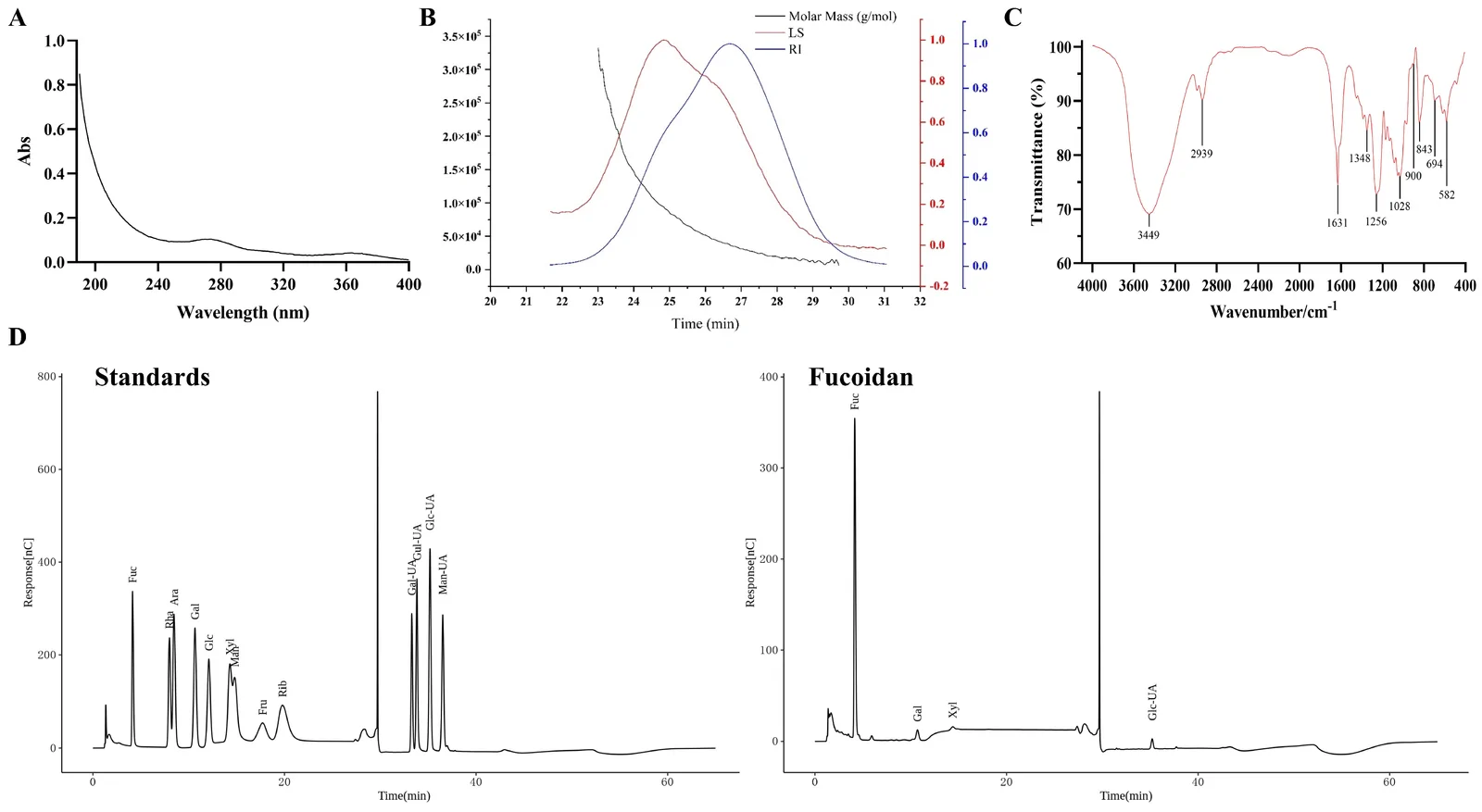
Structural characterization of fucoidan from *Fucus vesiculosus*. (**A**) UV-vis spectrum. (**B**) Molecular weight distribution determined by size-exclusion chromatography coupled with multi-angle laser light scattering and refractive index detection (SEC-MALLS-RI). (**C**) Fourier-transform infrared spectroscopy (FT-IR) spectrum. (**D**) Monosaccharide composition analyzed by high-performance anion-exchange chromatography with pulsed amperometric detection (HPAEC-PAD).

The purity and structural characteristics of the fucoidan were systematically characterized. The UV spectrum of fucoidan showed no obvious peaks at 260 nm and 280 nm (Figure 1A), indicating the absence of detectable nucleic acids and proteins, which confirmed the high purity of the sample. The molecular weight and its distribution were analysed by SEC-MALLS-RI (Figure 1B). The molecular weight (Mw) of fucoidan was estimated to be 54.65 kDa with a polydispersity index (Mw/Mn) of 1.60, indicating a relatively narrow molecular weight distribution. The content of the sulphate residue was determined as 47.4 ± 3.2%.

The functional groups and chemical bonds of fucoidan were characterised by FT-IR spectroscopy (Figure 1C). The broad absorption peak at 3449 cm^−1^ was assigned to the O-H stretching vibration, and the peak at 2939 cm^−1^ was attributed to the C-H stretching vibration of the sugar ring. The band at 1631 cm^−1^ was assigned to the bending vibration of bound water and asymmetric stretching vibration of carboxylate group (COO^−^). The absorption peak at 1256 cm^−1^ was characteristic of asymmetric stretching vibration of the sulphate groups (S=O), and the peak at 843 cm^−1^ was assigned to the bending vibration of C-O-S, which is characteristic of the sulphate groups substituted at the axial C-4 position of the fucose residues. In addition, the peak at 1028 cm^−1^ was attributed to the stretching vibrations of C-O-C and C-O-H of the pyranose ring. The spectral features match the typical profile of a sulfated polysaccharide, confirming the structural identity of the fucoidan.

Monosaccharide composition was determined by HPAEC-PAD. The chromatographic profile (Figure 1D) showed that the fucoidan was mainly composed of fucose (Fuc, 91.51%), followed by small amounts of galactose (Gal, 3.54%), glucuronic acid (Glc-UA, 3.52%) and xylose (Xyl, 1.43%) according to the retention time of standard monosaccharides. This sulfated and fucose-enriched macromolecular profile provides the structural basis for subsequent biological evaluation.

### 2.2. Fucoidan Alleviates Systemic Toxicity and Cognitive Dysfunction in Adenine-Induced CKD Mice

**Figure 2:**
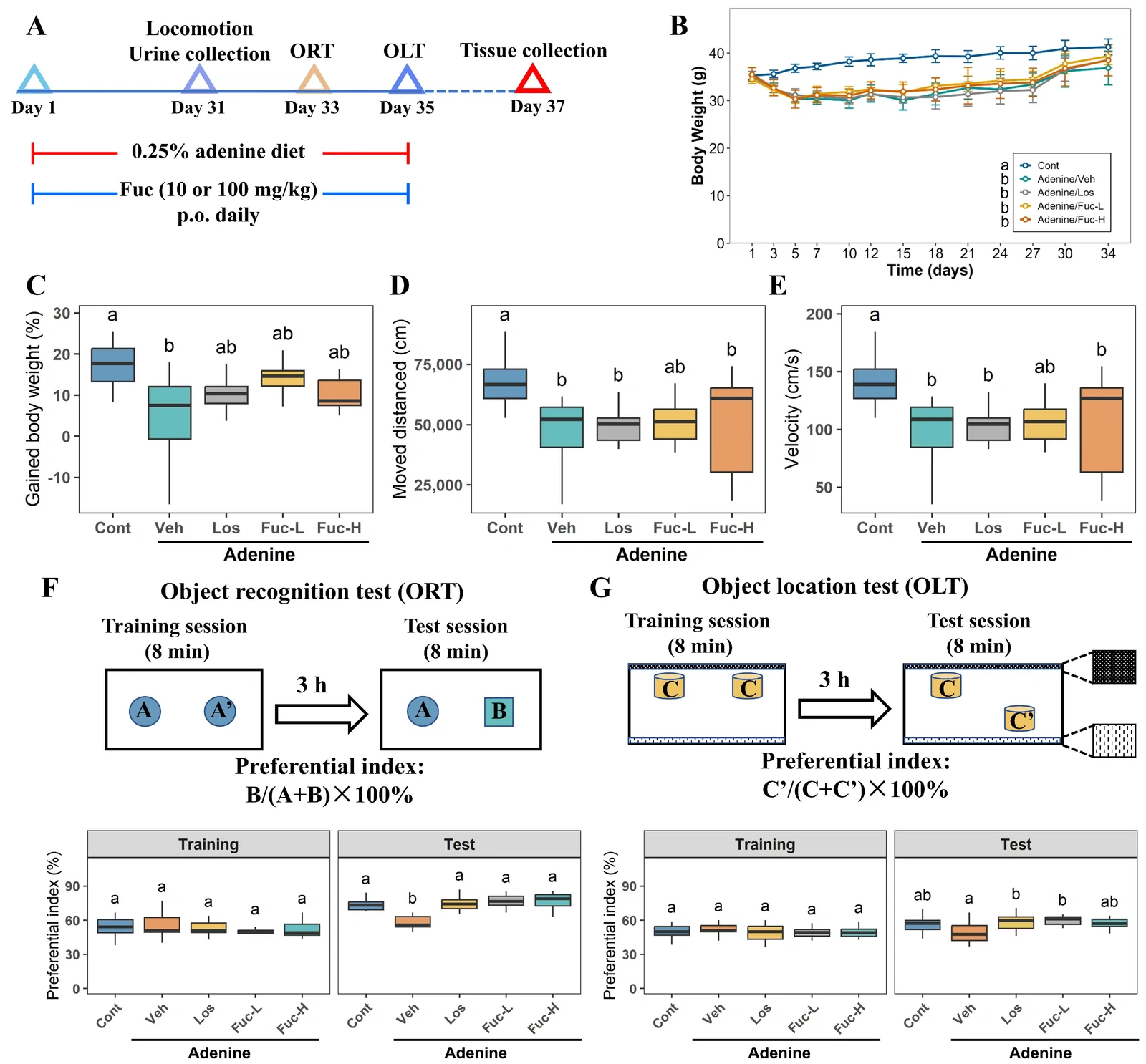
Fucoidan ameliorates adenine-induced toxicity and cognitive dysfunction in CKD mice. (**A**) Schematic illustration of the experimental design. Mice were fed a 0.25% adenine diet to induce CKD and orally administered with fucoidan (10 mg/kg for Fuc-L, or 100 mg/kg for Fuc-H) or the positive control losartan (20 mg/kg for Los) daily. (**B**) Dynamic changes in body weight during the experimental period. Body-weight data are presented as mean ± SD and were analysed using two-way repeated-measures ANOVA with group and time as factors. (**C**) Percentage of gained body weight at the end of the experiment, analysed using the Kruskal–Wallis test followed by Dunn’s multiple-comparisons test. (**D**) Total distance moved in the locomotion test. (**E**) Mean velocity in the locomotion test, analysed using one-way ANOVA followed by Tukey’s multiple-comparisons test. (**F**) Schematic diagrams and preferential index of the object recognition test (ORT). (**G**) Schematic diagrams and preferential index of the object location test (OLT). Training and test data in panels (**F**,**G**) were analysed separately using one-way ANOVA followed by Tukey’s multiple-comparisons test. For panels (**B**–**G**), *n* = 10 per group. Panels (**C**–**G**) are presented as box plots; the centre line indicates the median, boxes indicate the interquartile range. Groups with different lowercase letters indicate statistically significant differences (*p* < 0.05).

To investigate the protective effects of fucoidan against CKD and its complications, we established a mouse model of CKD induced by adenine, and the experimental timeline is shown in Figure 2A. Compared with the Control group, the mice that were fed with 0.25% adenine diet (Veh group) displayed systemic toxicity, as reflected by the rapid loss of body weight in the early modelling process and impaired overall weight gain (Figure 2B,C). Intervention with either fucoidan or losartan did not completely reverse weight loss, but did effectively stabilise body weight, suggesting partial protection against physical decline induced by CKD.

Basal locomotor activity was assessed to rule out the possibility that subsequent cognitive evaluations were confounded by uremia-induced motor deficits. As shown in Figure 2D,E, adenine exposure significantly reduced both the total distance moved and the mean velocity of the mice. Administration of Fuc-L partially attenuated these reductions, indicating an amelioration of CKD-induced motor impairment.

The object recognition test (ORT) and object location test (OLT) were employed to evaluate hippocampus-dependent recognition memory. In this context, the ORT measures the ability to discriminate a novel object (non-spatial component), whereas the OLT measures the ability to discriminate a novel object location (spatial component). Both tasks collectively assess recognition memory, a critical domain that is affected in CKD. During the training sessions, mice in all groups exhibited no significant object or spatial preference, with the preferential index maintained at approximately 50% (Figure 2F,G). However, in the ORT test session (Figure 2F), the Veh group failed to exhibit a preference for the novel object, indicating CKD-induced recognition memory deficits. Treatment with Fuc-L and Fuc-H restored the preferential index to levels comparable to those of the Control group. Consistent results were observed in the OLT (Figure 2G), where adenine-induced spatial memory impairment was significantly attenuated by fucoidan intervention. The positive control, losartan, similarly improved cognitive performance. Collectively, these behavioral assessments indicate that oral administration of fucoidan attenuates CKD-associated recognition memory, preserving both recognition and spatial memory.

### 2.3. Fucoidan Ameliorates Renal Structural Damage, Tubulointerstitial Fibrosis, and Oxidative Stress in CKD Mice

**Figure 3:**
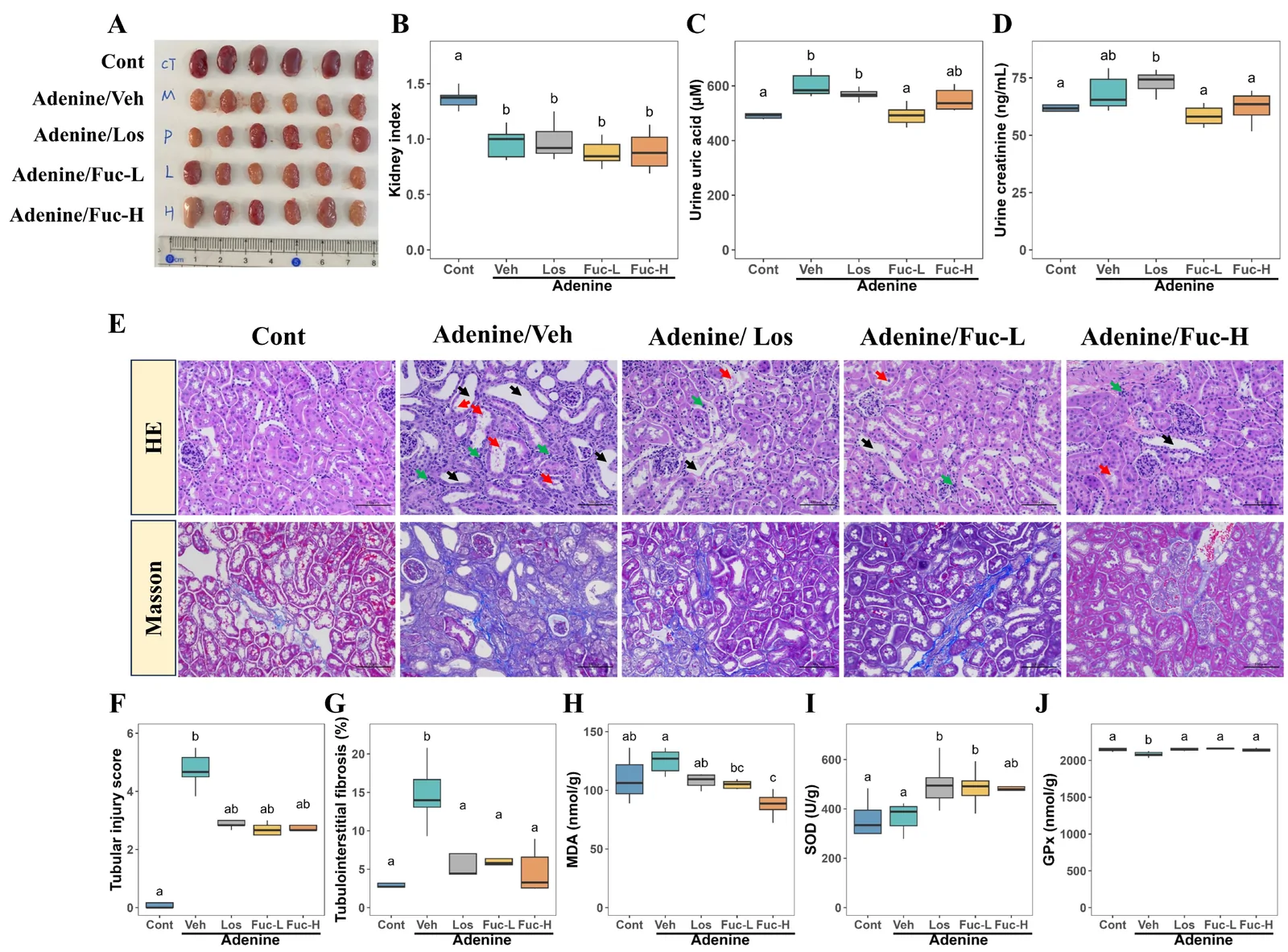
Fucoidan ameliorates renal structural damage, tubulointerstitial fibrosis, and oxidative stress in adenine-induced CKD mice. (**A**) Representative macroscopic images of kidney morphology. (**B**) Kidney index (Kidney weight (g)/body weight (g)). (**C**,**D**) Levels of urine uric acid (**C**) and urine creatinine (**D**), *n* = 6 per group. (**E**) Representative micrographs of HE staining (top row) and Masson’s trichrome staining (bottom row) of kidney tissues (Scale bar = 100 μm). Black arrows indicate tubular dilatation; Red arrows indicate epithelial cell desquamation or debris; Green arrows indicate inflammatory cell infiltration. (**F**,**G**) Quantitative analysis of tubular injury score (**F**) and tubulointerstitial fibrosis (**G**) based on histological staining, *n* = 5 per group. (**H**–**J**) Renal oxidative stress markers malondialdehyde (MDA) (**H**), superoxide dismutase (SOD) (**I**), and glutathione peroxidase (GPx) (**J**), *n* = 6 per group. Groups with different lowercase letters indicate statistically significant differences (*p* < 0.05). Kidney index (**B**), urinary uric acid (**C**), urinary creatinine (**D**), tubulointerstitial fibrosis (**G**), MDA (**H**), and SOD (**I**) were analysed using one-way ANOVA followed by Tukey’s multiple-comparisons test. Tubular injury score (**F**) and GPx (**J**) were analysed using the Kruskal–Wallis test followed by Dunn’s multiple-comparisons test.

Since the cognitive dysfunction in this model is driven by secondary uremic complications, we evaluated the ameliorative effects of fucoidan on the kidney, the primary target organ of CKD. On the macroscopic level, kidneys in the Control group exhibited a smooth surface and healthy reddish-brown color (Figure 3A). In contrast, kidneys from the Veh group appeared pale and granular, which are characteristic hallmarks of crystalline nephropathy and chronic renal injury. Fucoidan treatment attenuated these morphological abnormalities. Although the kidney index showed no obvious change relative to the Control group (Figure 3B), functional biochemical markers indicated effective renal protection. Specifically, the adenine-induced alterations in urine uric acid (Figure 3C) and urine creatinine (Figure 3D) were attenuated by fucoidan administration, with the Fuc-L group exhibiting the most pronounced mitigation.

To further assess structural protection at the microscopic level, histological evaluations were performed with HE and Masson’s trichrome staining (Figure 3E). HE staining revealed severe disruption of renal architecture in the Veh group, characterised by tubular dilatation, epithelial cell desquamation, and inflammatory cell infiltration. Both fucoidan treatment and losartan ameliorated these pathological lesions, which corresponded to a decreased tubular injury score (Figure 3F). In addition, Masson’s trichrome staining showed abundant blue-stained collagen fibres in the tubulointerstitium of the Veh group. Quantitative analysis confirmed a significant increase in the tubulointerstitial fibrotic area following adenine exposure (Figure 3G). Fucoidan treatment effectively suppressed the pathological accumulation of extracellular matrix. In particular, the Fuc-L group exhibited partial mitigation of fibrosis, whereas the Fuc-H more markedly restricted collagen deposition, restoring the tubulointerstitial architecture to a state comparable to that of the Control group.

Renal fibrosis is often associated with local redox imbalance, thus we assessed oxidative stress and antioxidant capacity in the kidney. Adenine exposure did not significantly increase renal malondialdehyde (MDA) levels compared with the Control group. However, both Fuc-L and Fuc-H significantly reduced renal MDA levels compared with the Veh group, with Fuc-H producing the greatest reduction (Figure 3H), indicating attenuation of local lipid peroxidation. Regarding antioxidant capacity, Fuc-L significantly increased superoxide dismutase (SOD) activity compared with the Veh group, whereas Fuc-H produced a modest increase that did not reach statistical significance (Figure 3I). Adenine exposure also significantly reduced the activity of glutathione peroxidase (GPx), whereas both Fuc-L and Fuc-H restored GPx activity to levels statistically comparable to that of the Control group (Figure 3J). Together, these results suggest that fucoidan may partially protect the kidney from injury by attenuating lipid peroxidation and supporting endogenous antioxidant defenses, thereby improving the local redox microenvironment and potentially contributing to the alleviation of tubulointerstitial fibrosis.

### 2.4. Fucoidan Alleviates Peripheral and Hippocampal Oxidative Stress and Inflammation in Adenine-Induced CKD Mice

**Figure 4:**
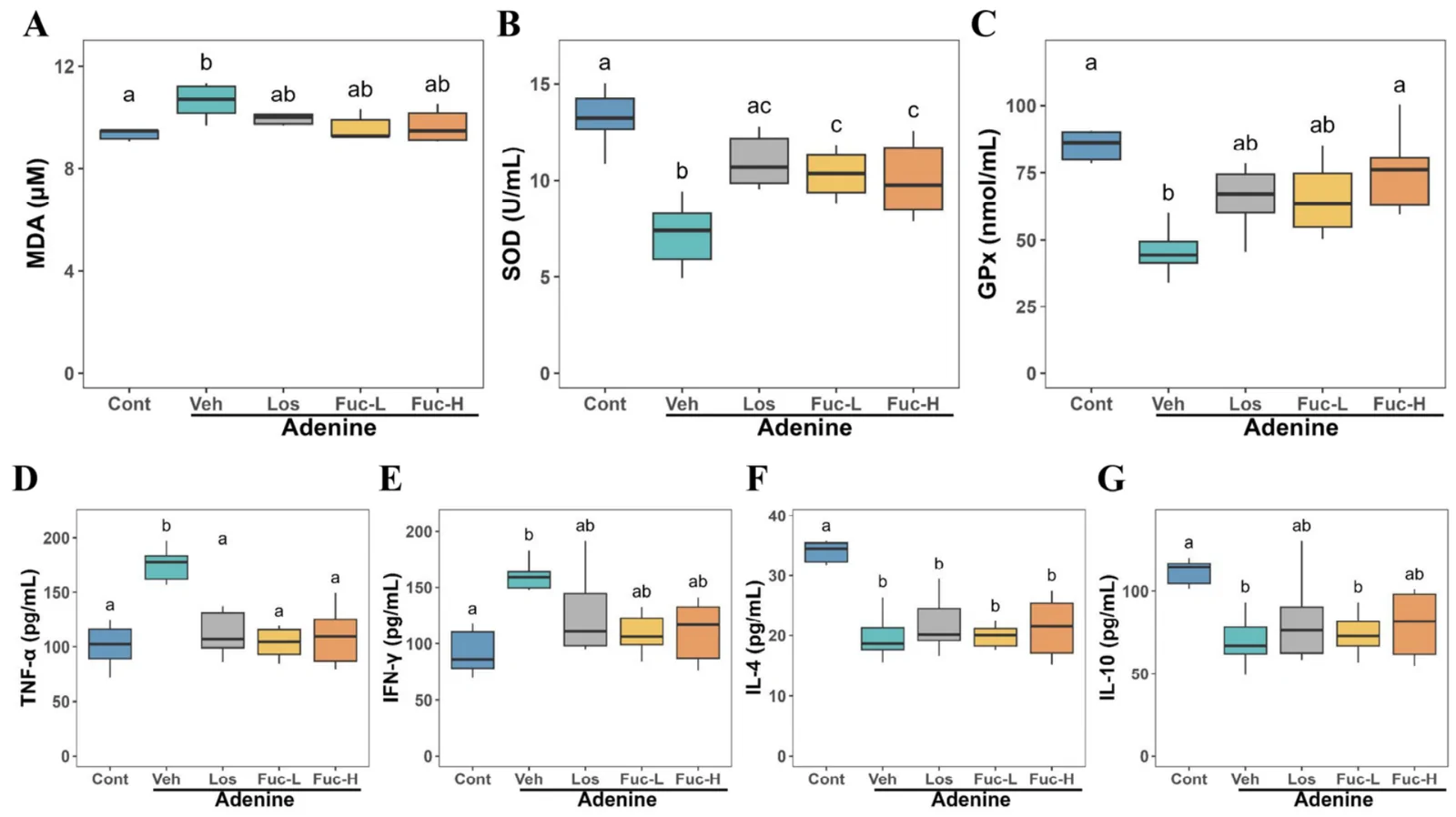
Fucoidan alleviates systemic oxidative stress and inflammation in adenine-induced CKD mice. (**A**–**C**) Serum levels of oxidative stress markers malondialdehyde (MDA) (**A**), superoxide dismutase (SOD) (**B**), and glutathione peroxidase (GPx) (**C**), *n* = 6 per group. (**D**–**G**) Systemic concentrations of pro-inflammatory and anti-inflammatory cytokines tumor necrosis factor-alpha (TNF-α) (**D**), interferon-gamma (IFN-γ) (**E**), interleukin-4 (IL-4) (**F**), and IL-10 (**G**), *n* = 6 per group. Data are presented as box plots showing the median and interquartile range with individual data points. Groups with different lowercase letters indicate statistically significant differences (*p* < 0.05). Serum SOD (**B**), TNF-α (**D**), IFN-γ (**E**), IL-4 (**F**), and IL-10 (**G**) were analysed using one-way ANOVA followed by Tukey’s multiple-comparisons test. Serum MDA (**A**) and GPx (**C**) were analysed using the Kruskal–Wallis test followed by Dunn’s multiple-comparisons test.

Since renal failure leads to the accumulation of uremic toxins in the bloodstream, causing systemic toxicity that can affect the central nervous system, we further studied circulating markers of oxidative stress and inflammation. As shown in Figure 4A, lipid peroxidation (MDA) levels were significantly elevated in the Veh group compared with the Control group, whereas the Los, Fuc-L, and Fuc-H groups showed intermediate levels that did not differ significantly from either group. Moreover, the endogenous antioxidant defense system was weakened after adenine exposure. The key antioxidant enzymes SOD (Figure 4B) and GPx (Figure 4C) showed significantly decreased circulating activities in the Veh group, suggesting systemic oxidative exhaustion. After fucoidan administration, these antioxidant capacities showed marker-dependent responses. Both Fuc-L and Fuc-H significantly increased SOD activity compared with the vehicle group. Similarly, Fuc-H effectively restored GPx activity to levels statistically comparable to those of the Control group. Although certain dose groups exhibited only intermediate trends that did not reach statistical significance, the overall trend indicates that fucoidan intervention contributes to the partial restoration of systemic redox homeostasis.

Inflammation is a key feature of the uremic microenvironment and a major contributor to secondary neuroinflammation. Pro-inflammatory cytokine analysis showed that adenine exposure induced a systemic inflammatory response, as evidenced by significant increases in serum concentrations of TNF-α (Figure 4D) and IFN-γ (Figure 4E). Fucoidan intervention attenuated this inflammatory response, with both doses significantly reducing TNF-α concentrations to near-baseline levels. For IFN-γ, both fucoidan-treated groups were not significantly different from either the Veh or Control group, although a downward trend relative to the Veh group was observed. Immune homeostasis was further assessed by measuring anti-inflammatory cytokines. No significant differences were observed in serum IL-4 levels after Fuc treatment (Figure 4F). Although Fuc-H did not significantly increase IL-10 concentrations relative to the Veh group, it produced an upward trend (Figure 4G). Together, these results suggest that fucoidan attenuates systemic oxidative stress and inflammation induced by uremia, potentially reducing the pathological signalling from injured kidneys to the brain.

**Figure 5:**
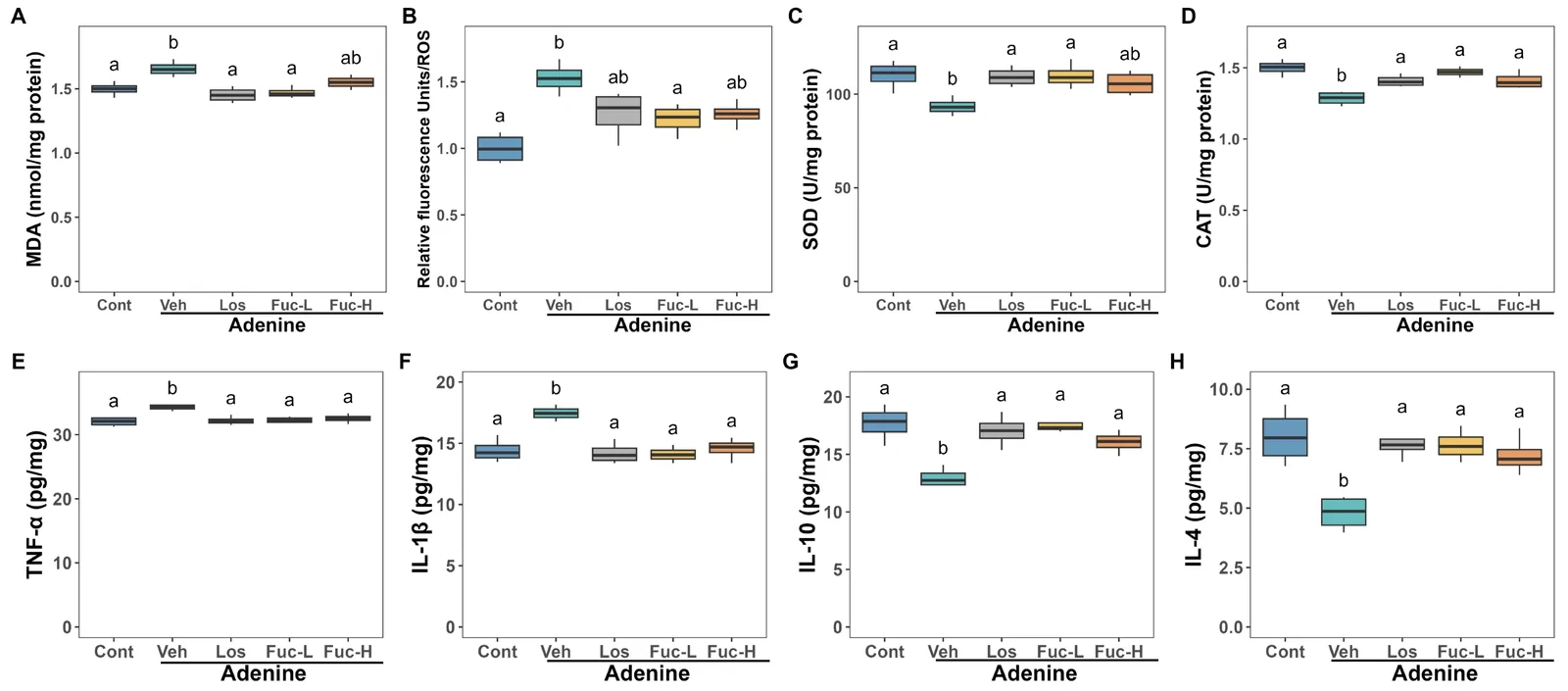
Fucoidan alleviates hippocampal oxidative stress and neuroinflammation in adenine-induced CKD mice. (**A**–**D**) Levels of hippocampal oxidative stress markers: (**A**) malondialdehyde (MDA), (**B**) reactive oxygen species (ROS), (**C**) superoxide dismutase (SOD), (**D**) catalase (CAT). (**E**–**H**) Levels of hippocampal inflammatory cytokines: (**E**) tumor necrosis factor-alpha (TNF-α), (**F**) interleukin-1β (IL-1β), (**G**) interleukin-10 (IL-10), and (**H**) interleukin-4 (IL-4). Data are presented as box plots showing the median and interquartile range with individual data points (*n* = 4 per group). Groups with different lowercase letters indicate statistically significant differences (*p* < 0.05). Group differences were analysed using one-way ANOVA followed by Tukey’s multiple-comparisons test.

To investigate whether the systemic protective effects of fucoidan translate into local improvements in the brain, we measured oxidative stress markers and inflammatory cytokine levels in hippocampal tissues. As shown in Figure 5A–D, adenine exposure significantly increased hippocampal MDA and ROS levels, while markedly reducing the activities of antioxidant enzymes SOD and CAT, indicating an oxidative shift in the brain. Fucoidan intervention effectively reversed these changes, restoring antioxidant enzyme activities and reducing lipid peroxidation to levels comparable to those of the Control group. Furthermore, the hippocampal inflammatory profile (Figure 5E–H) revealed that CKD mice exhibited elevated levels of pro-inflammatory cytokines TNF-α and IL-1β, alongside decreased levels of anti-inflammatory cytokines IL-10 and IL-4. Treatment with fucoidan significantly attenuated the elevation of TNF-α and IL-1β and partially restored IL-10 and IL-4 levels. Collectively, these results provide direct evidence that fucoidan ameliorates oxidative stress and neuroinflammation in the hippocampus, a key brain region for recognition memory, thereby supporting the observed behavioral improvements.

### 2.5. Fucoidan Modulates Gut Microbiota Composition and Correlates with Systemic Oxidative-Inflammatory Markers

**Figure 6:**
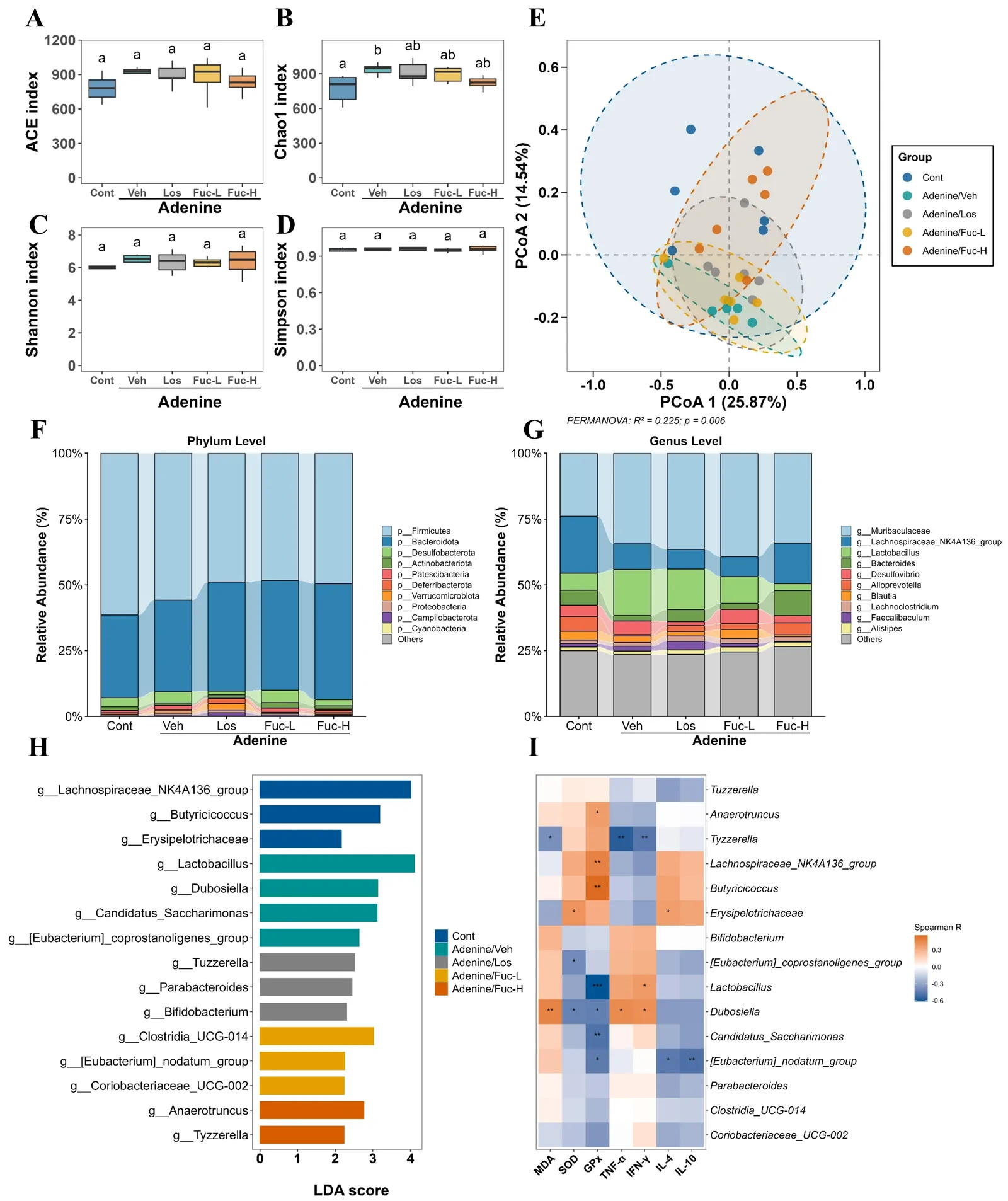
Fucoidan modulates gut microbiota composition and correlates with systemic oxidative-inflammatory markers. Alpha-diversity indices of the gut microbial community, including (**A**) ACE index, (**B**) Chao1 index, (**C**) Shannon index, and (**D**) Simpson index. (**E**) Principal Coordinates Analysis (PCoA) plot based on Bray–Curtis distance illustrating beta-diversity structural shifts. (**F**,**G**) Taxonomic distribution of relative abundance at the phylum (**F**) and genus (**G**) levels. (**H**) Linear discriminant analysis Effect Size (LEfSe) identifies distinct biomarker taxa among groups with an LDA score exceeding 2.0. (**I**) Spearman correlation matrix between the top 15 differential microbial genera and systemic oxidative/inflammatory markers, *n* = 6 per group. Red indicates a positive correlation, while blue indicates a negative correlation. Data in panels (**A**–**D**) are presented as box plots, in which the centre line indicates the median, boxes indicate the interquartile range. ACE (**A**) and Chao1 (**B**) were analysed using one-way ANOVA followed by Tukey’s multiple-comparisons test; Shannon (**C**) and Simpson (**D**) were analysed using the Kruskal–Wallis test followed by Dunn’s multiple-comparisons test. Groups with different lowercase letters indicate statistically significant differences (*p* < 0.05). The following notations are used for the heatmap: * *p* < 0.05, ** *p* < 0.01 and *** *p* < 0.001.

The low oral bioavailability of macromolecular polysaccharides indicates the gastrointestinal tract as the main site of action [29]. Thus, the modulatory effect of fucoidan on the gut microbiome was investigated by 16S rRNA gene sequencing. For alpha diversity (Figure 6A–D), ACE, Shannon and Simpson indices showed no significant differences among the groups. In contrast, the Chao1 index (Figure 6B), an estimator of community richness, was significantly increased after adenine induction (Veh group). Fucoidan treatment attenuated this elevation and brought the index closer to Control levels. The microbial communities were spatially separated according to principal coordinate analysis (PCoA) of beta diversity (Figure 6E). The Veh group was distinctly separated from the Control group, indicating CKD-associated microbial alterations in the gut microbiota. Intervention with both doses of fucoidan (Fuc-L and Fuc-H) shifted the microbiota clusters towards the trajectory of the Control group, indicating a partial restoration of the microbial community structure.

Taxonomic profiling further clarified specific microbial changes. At the phylum level, the communities (Figure 6F) were dominated by members of the *Firmicutes* and *Bacteroidota* in all groups. At the genus level (Figure 6G), the relative abundances of some populations were changed by adenine exposure and the subsequent treatment with fucoidan. LEfSe analysis was performed to identify the differential taxa driving these community changes (Figure 6H). The analysis showed that the Veh group was characterized by enrichment of some genera such as *Lactobacillus*. In contrast, the Control group exhibited enrichment of the *Lachnospiraceae_NK4A136_group*, a known SCFA producer [25,30]. Moreover, high-dose fucoidan treatment resulted in enrichment of specific taxa, including *Anaerotruncus* and *Tyzzerella*.

A Spearman correlation analysis was performed to explore possible associations between changes in the gut microbiota and systemic markers (Figure 6I). The *Lachnospiraceae_NK4A136_group* was positively correlated with GPx and negatively correlated with TNF-α. Conversely, *Lactobacillus* (enriched in the Veh group) was negatively correlated with GPx and IL-10 and positively correlated with IFN-γ. Notably, Tyzzerella, enriched by high-dose fucoidan, exhibited significant negative correlations with the pro-inflammatory markers TNF-α and IFN-γ. Taken together, these results indicate that fucoidan affects the intestinal microbial composition in adenine-induced CKD mice and these specific microbial changes correlate with systemic oxidative and inflammatory markers.

### 2.6. Fucoidan Modulates the Serum Metabolome and Is Associated with Lower Abundance of Uremic Toxins

**Figure 7:**
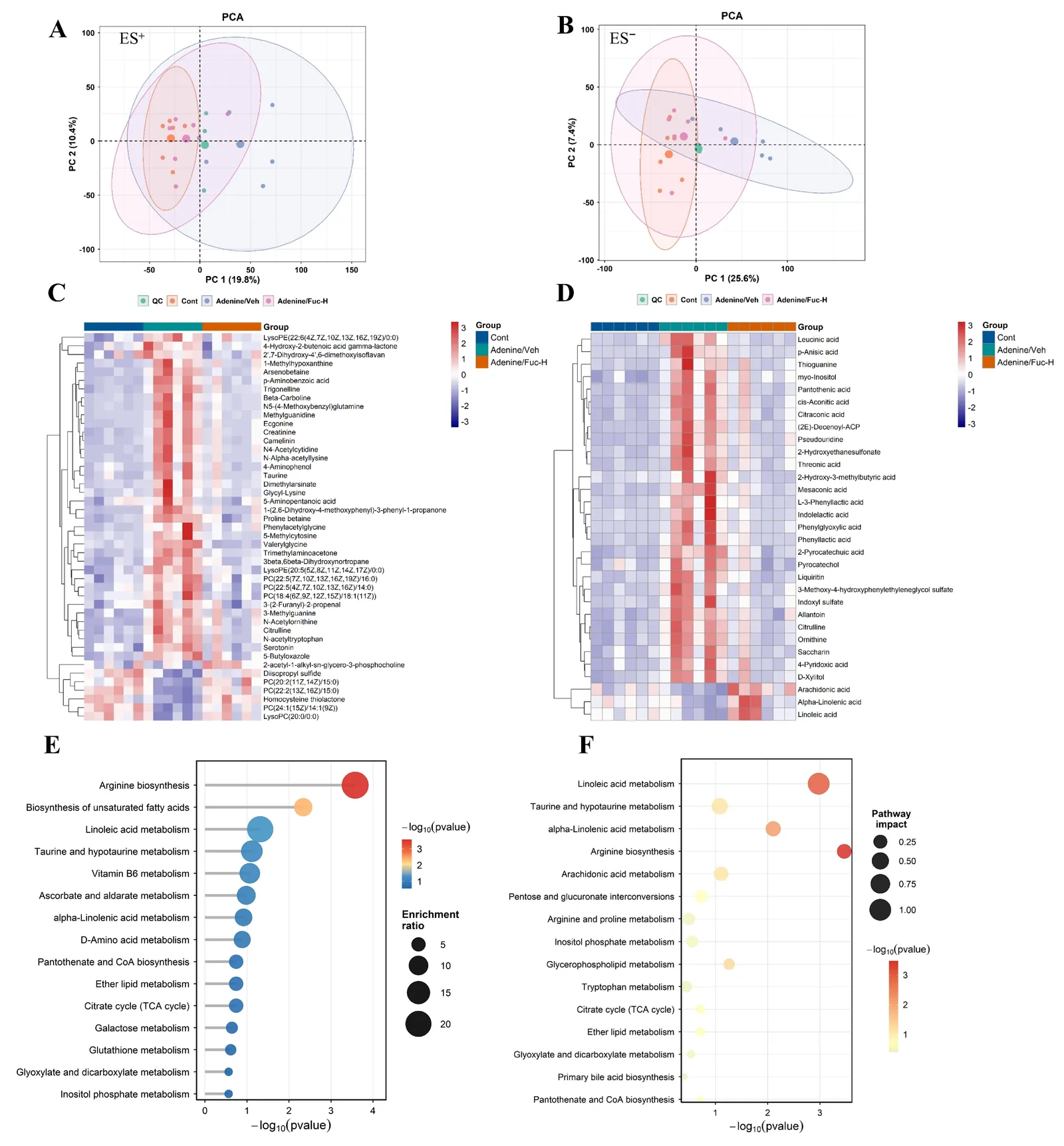
Fucoidan modulates the serum metabolome and is associated with lower abundance of uremic toxins. (**A**,**B**) PCA plots of the serum metabolome in positive ionization mode (ES^+^) (**A**) and negative ionization mode (ES^−^) (**B**). The tight clustering of Quality Control (QC) samples indicates robust instrument stability. Hierarchical clustering heatmaps of relative abundance of significantly differential serum metabolites in Control (Cont), Vehicle (Veh) and high-dose Fucoidan (Fuc-H) groups in ES^+^ (**C**) and ES^−^ (**D**) modes. Dot plot of metabolite set enrichment analysis (**E**) and bubble plot of pathway topology analysis (**F**) show the major biological pathways regulated by fucoidan, *n* = 6 per group (Cont, Veh, Fuc-H).

To elucidate the systemic changes accompanying gut microbiota modulation, non-targeted LC-MS metabolomics was employed to profile the serum metabolome. Unsupervised principal component analysis (PCA) (Figure 7A,B) revealed a distinct spatial separation between the Veh group and the Control group. The clustering of quality control (QC) samples within the PCA score plots indicated the stability and reproducibility of the analytical system. This metabolic deviation was further corroborated by supervised partial least squares-discriminant analysis (PLS-DA) (Figure S1A,B). Following intervention with Fuc-H, both the PCA and PLS-DA models exhibited a shift in the metabolic profiles, diverging from the Veh group trajectory and approaching the Control cluster. These results suggest that fucoidan partially restores the serum metabolic profile altered by adenine induction.

Heatmaps of hierarchical clustering were used to visualise the metabolic changes specific to the experimental groups (Figure 7C,D). The analysis revealed that the serum levels of creatinine and methylguanidine (Figure 7C) and indoxyl sulfate (Figure 7D) were increased in the Veh group, but were decreased by fucoidan intervention. Moreover, specific lipid metabolites, including phospholipids, such as several phosphatidylcholines (PCs) and lysophosphatidylcholine (LysoPC) (Figure 7C), and polyunsaturated fatty acids, such as arachidonic acid, alpha-linolenic acid and linoleic acid (Figure 7D), which were decreased in the Veh group, were elevated in the Fuc-H group.

To map these metabolic alterations to broader biological networks, the distinct differential metabolites from both ionization modes were integrated. A dual-approach pathway analysis, comprising metabolite set enrichment analysis (ORA) (Figure 7E) and pathway topology analysis (Figure 7F), was conducted. ORA results showed that fucoidan intervention significantly regulated the arginine biosynthesis pathway with the involvement of metabolites including arginine and citrulline. In addition, the linoleic acid metabolism pathway was also significantly enriched in the differential metabolites.

Lipid metabolism pathways—linoleic acid, arachidonic acid, and α-linolenic acid metabolism—yielded the highest pathway impact scores (Figure 7F). Nucleotide-related intermediates (e.g., N4-acetylcytidine) were also identified, consistent with adenine being a purine nucleobase. Collectively, fucoidan intervention was associated with shifts along three complementary axes: (i) uremic-toxin features; (ii) lipid metabolism; and (iii) nucleotide/amino-acid metabolism.

### 2.7. Multi-Omics Correlations Among Gut Microbiota, Serum Metabolites, and Oxidative-Inflammatory Markers

**Figure 8:**
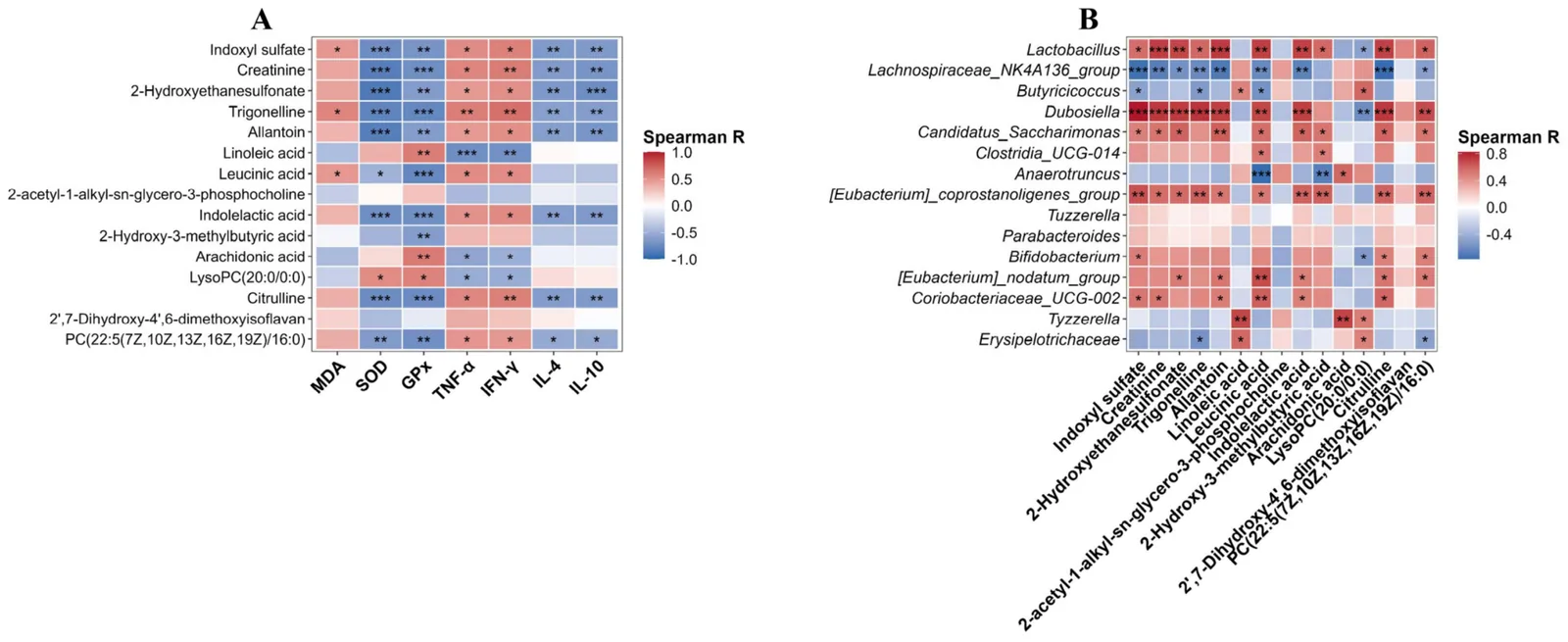
Multi-omics correlations among gut microbiota, serum metabolome and biochemical indices. (**A**) Spearman’s correlation heatmap of the correlations between top 15 differential serum metabolites and systemic biochemical indicators (oxidative stress and inflammatory cytokines). (**B**) Spearman’s correlation heatmap showing the host-microbiome crosstalk between the top 15 differential gut microbial genera and the key serum metabolites. The intensity of the colors represents the Spearman correlation coefficient, red for positive correlations and blue for negative correlations. Asterisks denote statistical significance: * *p* < 0.05, ** *p* < 0.01 and *** *p* < 0.001.

The relationships between gut microbial composition, circulating metabolites, and systemic oxidative and inflammatory markers were characterised by Spearman correlation analyses. Firstly, the correlation between the key serum differential metabolites and systemic biochemical indexes were evaluated (Figure 8A). Indoxyl sulfate, a gut-derived uremic toxin, was significantly negatively correlated with antioxidant enzymes SOD and GPx, and significantly positively correlated with pro-inflammatory cytokines including TNF-α and IFN-γ. Comparable correlation patterns were observed for renal function indicators (e.g., creatinine) and specific amino acid intermediates (e.g., citrulline). These statistical associations indicate that the levels of these circulating metabolites correlate with the markers of systemic oxidative stress and inflammation.

To explore the microbiota-metabolite crosstalk, the correlations of the genus level gut microbiota and serum metabolome were analysed (Figure 8B). The analysis revealed specific clustering patterns. The relative abundance of the genus *Lachnospiraceae_NK4A136_group* increased after fucoidan intervention and was significantly negatively correlated with a subset of metabolites including indoxyl sulfate, creatinine and indolelactic acid. In contrast, taxa such as *Dubosiella* and *Lactobacillus*, enriched in the vehicle-treated group, were positively correlated with these same metabolites. This correlation analysis reveals a synchronized relationship among specific intestinal bacterial genera, circulating uremic metabolites, and systemic oxidative/inflammatory indices. These multi-omics associations are consistent with possible involvement of the gut microenvironment in the phenotypic effects observed after fucoidan treatment.

## 3. Discussion

CKD is increasingly recognized as a systemic disorder that leads to progressive impairment of CNS function and manifests as clinical symptoms of uremic encephalopathy or CKD-associated cognitive impairment [3,6]. This inter-organ crosstalk is triggered by the build-up of uremic toxins, systemic inflammation and enhanced oxidative stress and contributes to neurovascular injury, blood–brain barrier disruption and neurotoxicity [5,31]. Here, we demonstrate that oral fucoidan administration from *Fucus vesiculosus* ameliorates renal structural damage and recognition memory deficits, and attenuates hippocampal oxidative stress and neuroinflammation, in adenine-induced CKD mouse model.

Furthermore, this study provides new insights into the systemic pharmacological behavior of bioactive macromolecules with limited oral bioavailability. Previously, we reported that fucoidan derived from *Laminaria japonica* could alleviate CKD-associated cognitive decline, an effect mediated via the modulation of microglial polarization and the GSK3β/Nrf2 signaling pathway in the brain [22]. However, given the high molecular weight and inherently limited oral bioavailability of fucoidan, the precise mechanisms by which this largely unabsorbable polysaccharide exerts protective effects on the remote central nervous system remained elusive. The present study offers a broader perspective on this phenomenon. By utilizing a highly sulfated fucoidan from *Fucus vesiculosus* and employing an integrative multi-omics approach, we shift our focus from localized neuroprotection to a microbiota-metabolite-host association network. Based on our current findings, we propose that the observed renoprotective and neuroprotective efficacies of fucoidan may not rely solely on direct local tissue interactions, but are rather associated with the upstream remodeling of the intestinal microbiota and the subsequent reduction in circulating uremic toxins. We emphasize that the multi-omics associations reported here are correlational and do not establish a definitive causal role of the microbiota-metabolite axis.

Losartan was included as a clinically established renoprotective agent, serving as a positive benchmark for renal endpoints rather than as a microbiota-targeted comparator. Its well-characterized hemodynamic and anti-fibrotic mechanisms make the observed cognitive improvement most parsimoniously interpreted as a downstream benefit of attenuated renal injury and reduced uremic burden [32,33]. Fucoidan, by contrast, is a high-molecular-weight, poorly absorbable polysaccharide; on bioavailability grounds, direct renal or central nervous system actions of the intact macromolecule are unlikely [23,24], pointing instead toward a gut-centered mode of action. However, poor oral absorption alone does not constitute proof of microbiota mediation—other gut-associated mechanisms, including effects on the intestinal barrier or local mucosal immunity, as well as potential contributions of absorbed low-molecular-weight fragments, cannot be excluded. The observation that a pharmacological agent (losartan) and a putatively gut-microbiota-modulating polysaccharide (fucoidan) both attenuate cognitive and renal phenotypes is therefore not contradictory. Rather, it suggests that distinct upstream interventions can converge on shared downstream nodes—reduced systemic uremic toxin accumulation, oxidative stress, and inflammation—to produce comparable phenotypic rescue. Given that losartan and fucoidan engage distinct upstream mechanisms—hemodynamic/anti-fibrotic versus putatively gut-microbiota-centered—yet converge on these shared downstream nodes, their co-administration could plausibly yield additive or synergistic benefit; combination strategies that pair conventional renoprotective agents with gut-microbiota- or antioxidant-targeted natural products are therefore an attractive, albeit unproven, therapeutic direction [33,34]. Nevertheless, whether losartan and fucoidan act synergistically, additively, or even redundantly remains to be established, and a dedicated combination study is a logical next step.

In the progression of CKD, impaired renal filtration capacity leads to the systemic accumulation of metabolic wastes, which subsequently exacerbates oxidative stress and inflammatory responses, driving progressive multi-organ damage [34,35,36]. Consistent with the well-characterized adenine-induced CKD model, which effectively recapitulates this systemic redox imbalance and inflammation [37], our study observed significant neurocognitive and renal structural impairments in vehicle-treated mice. Oral administration of fucoidan ameliorated recognition and spatial memory deficits, as evaluated by the ORT and OLT, respectively. Alterations in biochemical markers of renal function and histopathological evidence of tubular injury and tubulointerstitial fibrosis were also attenuated. These phenotypic improvements were accompanied by partial restoration of systemic redox homeostasis, as evidenced by increased circulating SOD activity following both fucoidan treatments and restoration of GPx activity by Fuc-H, although the reduction in circulating MDA did not reach statistical significance. Fucoidan also attenuated systemic inflammation, as demonstrated by a significant reduction in serum TNF-α, whereas IFN-γ showed only a nonsignificant downward trend. Neither dose restored IL-4 levels, while Fuc-H produced a nonsignificant upward trend in IL-10. Together, these findings suggest that fucoidan partially attenuates systemic uremic toxicity and may thereby reduce pathological kidney-to-brain signalling. In parallel, hippocampal biochemical analysis showed that adenine-induced increases in MDA and ROS, decreases in SOD and CAT activities, and the pro-inflammatory shift (elevated TNF-α and IL-1β with reduced IL-10 and IL-4) were attenuated by fucoidan. This pattern is consistent with current evidence that CKD-associated cognitive decline involves convergent effects of uremic toxins, oxidative stress, inflammation, and blood–brain barrier dysfunction [6,11,18,31]. It is also directionally consistent with our previous study in adenine-induced CKD mice, in which *Laminaria japonica*-derived fucoidan improved memory deficits and kidney- and hippocampus-associated oxidative-inflammatory signalling [22]. Given its oral administration and macromolecular nature, the gastrointestinal tract may contribute to the effects of fucoidan. These renal and systemic phenotypes are in line with the broad renoprotective actions documented for natural polysaccharides in various kidney-injury models [34], and the redox-inflammatory signature observed here is consistent with systemic alterations recently described by multi-omics profiling of the adenine-induced CKD model [37].

An additional observation was that the two fucoidan doses did not uniformly exhibit a monotonic dose–response relationship across all endpoints. For instance, Fuc-L appeared more effective in ameliorating alterations in urinary uric acid and creatinine, whereas Fuc-H exerted a more pronounced effect on tubulointerstitial fibrosis and systemic GPx activity. Notably, as the study was not designed for direct statistical comparisons between the two doses, no ranking of their relative efficacy is implied. This non-monotonic pattern is not uncommon for macromolecular polysaccharides and could potentially reflect dose-dependent differences in colonic fermentation dynamics, whereby varying polysaccharide loads selectively enrich distinct microbial taxa and, consequently, modulate different metabolic and signaling pathways [38,39,40]. The efficacy at the lower dose and the absence of overt adverse effects at the higher dose are encouraging, yet a definitive therapeutic window and safety profile will require dedicated dose-ranging and toxicological studies.

The gut microbiota plays an important role in the gut-kidney axis and is responsible for the endogenous production of uremic toxins and the regulation of systemic immunity [13,15]. Our 16S rRNA gene sequencing revealed that adenine-induced CKD disturbed normal gut microbial composition. The oral administration of fucoidan effectively modulated this dysbiosis. LEfSe analysis revealed an association between the disease state and enrichment of some taxa, especially *Lactobacillus* and *Dubosiella*. Correlation analysis also demonstrated that *Lactobacillus* was negatively correlated with the antioxidant enzyme GPx and positively correlated with the pro-inflammatory cytokine IFN-γ. In contrast, high-dose fucoidan intervention specifically enriched genera such as *Anaerotruncus* and *Tyzzerella*. Notably, *Tyzzerella* exhibited significant negative correlations with systemic pro-inflammatory markers (TNF-α and IFN-γ). These microbiota-associated patterns are broadly consistent with evidence that dietary polysaccharides can ameliorate CKD-related complications [41] and with reports of altered SCFA-producing and uremic-toxin-producing microbial populations in CKD [16]. However, the functional roles of individual taxa cannot be inferred from 16S rRNA gene sequencing alone. Targeted measurement of microbial metabolites and functional metagenomic analyses will be needed to test these proposed links.

Microbial compositional changes were accompanied by changes in the metabolome. We investigated the metabolic changes induced by fucoidan by merging untargeted serum metabolomic profiles from both positive and negative ionization modes. The analysis showed that the treatment with fucoidan was associated with lower relative abundance of protein-bound uremic toxins, especially a putatively annotated indoxyl sulfate feature. This gut-derived metabolite, generated by bacterial fermentation of dietary amino acids, has been demonstrated to cross the disrupted blood–brain barrier, promoting neuroinflammation and cognitive impairment [42,43]. Moreover, pathway enrichment analyses indicated that fucoidan primarily modulated the networks of lipid and amino acid metabolism. Metabolomic profiles, in particular, suggested a recovery of polyunsaturated fatty acids (PUFAs) such as arachidonic acid and linoleic acid that were depleted in the vehicle-treated CKD group. Systemic oxidative stress in progressive CKD induces the lipid peroxidation of circulating PUFAs, although systemic MDA levels did not reach statistical significance in our cohort, renal MDA was significantly lowered by Fuc-H. The restoration of these circulating lipid pools in fucoidan-treated mice provides a metabolic context for the concurrent attenuation of systemic inflammatory markers (e.g., TNF-α, IFN-γ). Meanwhile, the regulation of taurine and arginine biosynthesis pathways is in accordance with the observed improvement in endogenous antioxidant defenses (SOD, GPx). Collectively, the modulation of these specific metabolic networks mitigates the systemic dysregulation characteristic of progressive CKD [44,45]. The high pathway-impact scores for lipid metabolism and the foregrounding of indoxyl sulfate reflect distinct yet complementary facets of the same pathological cascade. Lipid pathway alterations (e.g., PUFA depletion and lipid peroxidation) are largely secondary to systemic oxidative stress, whereas indoxyl sulfate is proposed as a candidate gut-microbiota-derived bridging signal connecting microbial dysbiosis to host inflammatory responses. These two lines of evidence are complementary, highlighting both the broad impact and a candidate microbe-host axis. Overall, the uremic-toxin and lipid/amino-acid metabolic signatures identified here broadly align with serum metabolomic disturbances reported in CKD patients and models [44,45]. Nevertheless, because metabolite identities were putatively annotated in an untargeted analysis and the multi-omics relationships are correlational, the present findings do not establish causal links among individual metabolites, microbial taxa, and cognitive outcomes. Targeted metabolite quantification and intervention studies will be required to validate these candidate pathways.

Spearman’s correlation analysis was conducted to integrate the multi-level datasets, which revealed significant statistical associations between certain gut bacterial abundances and serum metabolite concentrations, as well as systemic oxidative and inflammatory markers. These associations suggest potential mechanisms underlying the effects of fucoidan; however, several limitations should be acknowledged. First, blood–brain barrier integrity was not directly assessed in our animals (e.g., by Evans Blue extravasation or tight-junction protein measurement). Second, the multi-agent anesthetic cocktail may affect the serum metabolome, although all groups were handled identically and the observed metabolic shifts correspond to established CKD metabolites. Third, the two fucoidan doses were not designed for head-to-head statistical comparison, and the non-monotonic dose–response is not fully resolved. Fourth, because losartan was used as a general renoprotective control rather than as a microbiota-targeted comparator, the present design cannot distinguish the microbiota-dependent effects of fucoidan from alternative microbiota-independent renoprotective actions. Future directions therefore include: fecal microbiota transplantation and specific microbial colonization or rescue models to causally validate the roles of the identified bacterial taxa; antibiotic depletion experiments; targeted quantification of indoxyl sulfate and related uremic toxins; direct assessment of blood–brain barrier integrity; dose-ranging studies paired with matched multi-omics; and anesthetic-metabolite exclusion as a refinement for future metabolomic studies. These experiments would move the present correlational findings toward causal mechanistic proof.

## 4. Materials and Methods

### 4.1. Materials and Reagents

Fucoidan from *Fucus vesiculosus* (Product No. F8190, purity ≥ 95%) and adenine (Product No. A8626, purity ≥ 99%) were obtained from Sigma-Aldrich (St. Louis, MO, USA). Losartan potassium tablets (50 mg/tablet) were supplied by Hangzhou Merck Sharp & Dohme Pharmaceutical Co., Ltd. (Hangzhou, China). All other chemicals and solvents used were of analytical or liquid chromatography-mass spectrometry (LC-MS) grade.

### 4.2. Structural Characterization of Fucoidan

Structural characterization of the fucoidan sample (spectral analysis, molecular weight determination and monosaccharide composition) was carried out according to our previously established methodology [46]. Briefly, characteristic UV and Fourier-transform infrared (FT-IR) spectra were measured using a Cary 60 UV spectrophotometer (Agilent Tech., Santa Clara, CA, USA) and a TENSOR 27 FT-IR spectrometer (Bruker, Rheinstetten, Germany). The molecular weight distribution was measured by size-exclusion chromatography coupled with a DAWN HELEOS-II multi-angle laser light scattering (Wyatt Tech, Santa Barbara, CA, USA) and refractive index detection (SEC-MALLS-RI, Optilab T-rEX, Wyatt Tech, Santa Barbara, CA, USA) and an UltiMate 3000 HPLC system (Thermo Fisher Scientific, Waltham, MA, USA) equipped with two tandem-column (Shodex OH-pak SB-803 and 805, Tokyo, Japan). Complete acid hydrolysis was performed for monosaccharide profiling and the composition of monosaccharides was determined by high-performance anion-exchange chromatography with pulsed amperometric detection (HPAEC-PAD) on a Dionex CarboPac PA-20 column (Thermo Fisher Scientific, Waltham, MA, USA).

### 4.3. Animals and Experimental Design

All animal care procedures and experimental protocols were conducted in strict compliance with the guidelines of the Animal Committee of Guangdong Ocean University (Approval No.: SYXK2021-0025). Fifty specific pathogen-free male ICR mice (8 weeks old, weighing 34 ± 2 g) were purchased from Guangdong Zhiyuan Biomedical Technology Co., Ltd. (Guangzhou, China). Mice were maintained under controlled conditions (temperature 22 ± 2 °C, humidity 55 ± 10%, 12 h dark/light cycle) and given standard SPF diet and purified water *ad libitum*. After 7 days of acclimation, the mice were randomly assigned into five groups (*n* = 10 for each group) including Normal Control (Cont), CKD Model Vehicle (Veh), Low-dose Fucoidan (Fuc-L), High-dose Fucoidan (Fuc-H) and Positive Control (Losartan, Los). Behavioral and body-weight endpoints were assessed in all ten mice per group. After euthanasia, four to six mice per group were allocated to serum and renal biochemical assays, histological assessment, 16S rRNA gene sequencing, serum metabolomics, and hippocampal biochemical analyses. This subset was chosen to ensure that all samples and their technical replicates could be processed simultaneously on a single assay plate, thereby minimizing inter-assay variability, while also maintaining cost-effectiveness. Importantly, the selection of these animals per group was performed randomly. Furthermore, to ensure consistency across multi-omics correlation analyses, the same six animals per group were used for 16S rRNA gene sequencing, serum metabolomics, and serum biochemical marker measurements.

The CKD model was induced by feeding the mice with a powdered diet containing 0.25% adenine in the four experimental groups, while the Cont group was fed a standard diet. Simultaneously, intervention strategies were given by daily intragastric gavage using a standard stainless steel feeding needle for 30 consecutive days. The Fuc-H and Fuc-L groups were administered fucoidan at doses of 100 mg/kg/day and 10 mg/kg/day, respectively. The Los group was given losartan suspension at a dose of 20 mg/kg/day. The fucoidan doses were selected based on a comprehensive literature survey of in vivo fucoidan studies, in which effective doses in rodent models typically range from 10 to 500 mg/kg/day. Our previous study on *Laminaria japonica*-derived fucoidan demonstrated that oral administration at 100 and 200 mg/kg/day significantly alleviated adenine-induced renal injury in mice [22]. Therefore, we chose 100 mg/kg/day as the high dose (Fuc-H) based on this validated efficacy, and 10 mg/kg/day as the low dose (Fuc-L) to explore potential dose-dependent effects. These values fall within the range of oral fucoidan doses (250–4400 mg/day) that have been evaluated in human clinical trials with reported safety and tolerability. Losartan was administered at 20 mg/kg/day as a positive control for renoprotection, a dose equivalent to the standard clinical human range of 50–100 mg/day. Cont and Veh groups received a comparable volume (5 mL/kg body weight) of distilled water. Body weights were monitored every other day for dynamic adjustment of gavage volumes.

### 4.4. Behavioral Assessments

After the 30-day intervention, locomotor and recognition memory were assessed in a quiet, dimly lit room. On testing days, drug administration was delayed until 1 h after the completion of each session. All behavioral procedures were conducted in accordance with previously validated protocols [47] with minor modifications.

Basal locomotor activity was evaluated using an open field apparatus (40 × 40 × 40 cm). Each mouse was placed gently at the center of the floor and allowed to explore freely for 5 min. The total distance moved and mean velocity were recorded and analyzed using the Shanghai Xinruan tracking software (Version 2.0). To eliminate olfactory cues from previous animals, the inner walls and floor of the apparatus were thoroughly cleaned with 5% ethanol solution between each trial.

The ORT was performed to assess non-spatial recognition memory. During the training phase, two identical objects (A and A′) were placed in the open field, aligned in a row at 4 cm from the left and right walls, respectively. Each mouse was placed into the apparatus facing the wall and allowed to explore the two objects for 8 min. The number of explorations for each object (tA and tA′) was recorded. After a 3-h retention interval, one of the objects (A′) was replaced with a novel object (B), and the mouse was reintroduced into the apparatus for another 8-min test session. The number of explorations for the familiar object (tA) and the novel object (tB) was recorded. The preferential index was calculated as: Preferential index = tB/(tA + tB).

The OLT was performed 48 h after the completion of the ORT to evaluate spatial recognition memory. A completely new set of objects (C and C′, differing in shape, color, and texture from those used in the ORT) was used. During the training phase, two identical objects (C and C′) were placed in the open field, aligned in a row at 4 cm from one side wall, with distinct patterns affixed to the side walls to provide spatial cues. Each mouse was placed into the apparatus facing the wall and allowed to explore the two objects for 8 min. The number of explorations for each object was recorded. After a 3-h retention interval, one object (C′) was moved to the diagonally opposite corner (novel location), while the other object (C) remained in its original position. The mouse was reintroduced for another 8-min test session, and the number of explorations for the object at the familiar location (tC) and the object at the novel location (tC′) was recorded. The preferential index was calculated as: Preferential index = tC′/(tC′ + tC).

### 4.5. Sample Collection and Biochemical Analysis

Following behavioral testing, mice were deeply anesthetized with a cocktail containing xylazine hydrochloride (23 mg/kg), zolazepam (15 mg/kg), and salbutamol hydrochloride (15 mg/kg), following a previously established protocol [48]. Blood samples were collected from the abdominal aorta immediately after deep anesthesia was achieved, centrifuged to obtain serum, and stored at −80 °C.

Mice were then transcardially perfused with ice-cold 1× PBS to remove blood from the tissues. Kidneys and brains were rapidly harvested. The renal capsule was carefully stripped from each kidney, and the hippocampus was dissected from each brain on an ice-cold plate. The collected tissues were minced into small pieces and lysed using M-PER™ Mammalian Protein Extraction Reagent (#78501, Thermo Fisher Scientific, Waltham, MA, USA) supplemented with a protease inhibitor cocktail (1×, #78429, Thermo Fisher Scientific, Waltham, MA, USA). The tissue lysates were homogenized using a Saierte SRT-24 homogenizer (Xiamen, China). After incubation on ice for 30 min, the homogenates were centrifuged at 12,000× *g* for 10 min at 4 °C. The supernatants were collected, and total protein concentrations were quantified using the Pierce™ 660 nm Protein Assay Reagent (#22660, Thermo Fisher Scientific, Waltham, MA, USA), and the samples were subsequently diluted 10-fold for further analyses.

In renal tissue, MDA, SOD, and GPx were measured. In serum, MDA, SOD, GPx, TNF-α, IFN-γ, IL-4, and IL-10 were measured. In hippocampal homogenates, MDA, ROS, SOD, CAT, TNF-α, IL-1β, IL-10, and IL-4 were measured. All assays were performed using commercial kits according to the manufacturers’ instructions. Detailed catalog numbers and manufacturers of all kits are provided in Table S1.

### 4.6. HE and Masson Staining

Kidney tissues were fixed in 4% paraformaldehyde, dehydrated, embedded in paraffin, and sectioned at 4 μm thickness. Sections were then subjected to hematoxylin and eosin (HE) staining or Masson’s trichrome staining for histopathological examination. For the evaluation of renal tubular injury, HE-stained sections were examined under a light microscope at ×200 magnification. A semi-quantitative scoring system was applied as follows: normal renal tubules (0 points); obviously dilated renal tubules (1 point); flattened or swollen epithelial cells (1 point); renal brush border membrane injury (1 point); cell debris within the tubular lumen (2 points); tubular casts (2 points); and cell shedding and necrosis in the lumen in the absence of tubular casts and cell debris (1 point). The total score for each field was calculated by summing all applicable criteria. For each mouse, six randomly selected high-power fields (three from the renal cortex and three from the corticomedullary junction) were scored. The average score per mouse was calculated.

For the assessment of tubulointerstitial fibrosis, Masson’s trichrome staining was performed, in which collagen fibers appeared blue. For each group, five mice and six sections per mouse were analyzed. Images were captured at ×200 magnification, and the blue-stained fibrotic areas were quantified using ImageJ software (Version 1.51). The tubulointerstitial fibrotic area was calculated as the ratio of the blue-stained area to the total tissue area within each field, and the average percentage for each group was calculated.

### 4.7. 16S rRNA Gene Sequencing and Microbiota Analysis

Total genomic DNA was extracted from colonic fecal samples by GHFDE100 Extraction Kit (Guhe, Hangzhou, China). The concentration of DNA was measured with a NanoDrop 2000 spectrophotometer (Thermo Fisher Scientific, Wilmington, DE, USA), and its purity was checked using 1% agarose gel electrophoresis. The hypervariable V4 region of the bacterial 16S rRNA gene was amplified with universal primers, which were standardized and linked to Illumina-specific barcodes, using Phusion High-Fidelity PCR Master Mix (Thermo Scientific). Amplicons were purified and quantified with Qubit 2.0 and sequenced on Illumina NovaSeq platform.

Bioinformatics analyses were run using the QIIME2 pipeline. Raw reads were filtered for quality, denoised and clustered into Amplicon Sequence Variants (ASVs). Taxonomic annotation was performed using the SILVA 138 database. Downstream microbial community ecology data mining and visualization were performed using the *microeco* R package [49]. Specifically, alpha-diversity was estimated by Shannon, Simpson, Chao1 and ACE indices. Principal Coordinates analysis (PCoA) based on Bray–Curtis distances was performed to visualize the structural changes in beta-diversity. Significant taxonomic biomarkers responsible for the differences between groups were identified using linear discriminant analysis effect size (LEfSe) with an LDA score of >2.0.

### 4.8. Serum Untargeted Metabolomics Analysis

Serum samples (50 μL) were mixed with 200 μL of extraction solvent, which included an isotopically labeled internal standard, utilizing a 1:1 (*v*/*v*) ratio of acetonitrile to methanol. Following vortexing for 30 s and ultrasonication in an ice-water bath for 10 min, the homogenates were incubated at −40 °C for 1 h to precipitate proteins. After centrifugation at 12,000× *g* for 15 min at 4 °C, the supernatant underwent LC-MS/MS analysis. Quality control (QC) samples were prepared by pooling equal aliquots of all supernatants to monitor instrument stability.

Metabolic profiling was performed on a Vanquish UHPLC system coupled with a Q Exactive HFX mass spectrometer (Thermo Fisher Scientific, Waltham, MA, USA). Chromatographic separation was achieved on a UPLC BEH Amide column. The mobile phases consisted of 25 mmol/L ammonium acetate and 25 mmol/L ammonia in water (Phase A) and pure acetonitrile (Phase B). The MS/MS spectra were acquired under Information-Dependent Acquisition (IDA) mode under both positive (ES^+^) and negative (ES^−^) ionization modes. QC samples were injected at regular intervals throughout the analytical run to ensure instrument stability. After data acquisition, raw data processing, metabolite alignment and peak integration were performed. Changes in the metabolic profiles were statistically analysed using multidimensional statistics such as Principal Component Analysis (PCA) and Partial Least Squares-Discriminant Analysis (PLS-DA). Significantly differential metabolites were identified according to the following criteria: a Variable Importance in Projection (VIP) score ≥ 1 from the PLS-DA model, an absolute log2 fold change (|log2FC|) > 0.25, and a statistical significance of *p* < 0.05. Then hierarchical clustering heatmap was used to visualise the relative abundances of these differential metabolites. To uncover the underlying systematic mechanisms, the differential metabolites in ES^+^ and ES^−^ were pooled together. Metabolite Set Enrichment Analysis (Over Representation Analysis, ORA) and Pathway Topology Analysis were conducted with MetaboAnalyst 6.0 [50] to clarify the major biological pathways regulated by the intervention.

### 4.9. Statistical Analysis

Quantitative data are presented as mean ± standard deviation (SD) or as box plots showing the median and interquartile range. Statistical analysis were performed using R software (version 4.5.1). For comparisons among multiple groups, one-way analysis of variance (ANOVA) followed by Tukey’s multiple-comparisons test was used when ANOVA residuals were normally distributed and variances were homogeneous. When either assumption was not met, the Kruskal–Wallis test followed by Dunn’s multiple-comparisons test with Bonferroni adjustment was used. Data involving two independent factors were analyzed using two-way ANOVA followed by Tukey’s multiple-comparisons test, where appropriate. Spearman’s rank correlation analysis was used to assess associations among selected gut microbial genera, serum metabolites, and phenotypic indicators (oxidative stress and inflammatory cytokines). *p* < 0.05 was considered statistically significant. Statistically significant differences between multiple groups in graphical representations are denoted by different lowercase letters (*p* < 0.05), while asterisks are used to denote significance in correlation heatmaps (* *p* < 0.05, ** *p* < 0.01, *** *p* < 0.001).

## 5. Conclusions

In conclusion, the present study demonstrates that oral administration of *Fucus vesiculosus*-derived fucoidan attenuates systemic and hippocampal oxidative stress, inflammation, and improves recognition-memory performance in an adenine-induced CKD murine model. The findings suggest that these renoprotective and neuroprotective effects are associated with the modulation of the gut microbiota and a lower relative abundance of circulating, putatively annotated uremic-toxin features, including indoxyl sulfate. Collectively, these results support the potential application of fucoidan as a marine-derived intervention for mitigating CKD progression and its associated neurological complications, although the observed microbiota–metabolite–host associations are correlational and require further causal validation.

## Data Availability

The original contributions presented in this study are included in the article/Supplementary Materials. Further inquiries can be directed to the corresponding author.

## Supplementary Materials

The following supporting information can be downloaded at https://www.mdpi.com/article/10.3390/md24090323/s1, Figure S1: Partial least squares-discriminant analysis (PLS-DA) of serum metabolomic profiles. (A,B) Score plots illustrate the spatial separation of metabolic phenotypes among theexperimental groups in positive ionization mode (ES^+^) (A) and negative ionization mode (ES^−^) (B); Table S1: The detailed information of reagents used for biochemical analysis.
