# Supplementary material for: Fucus vesiculosus-Derived Fucoidan Mitigates Recognition Memory Deficits in Association with Modulation of the Microbiota–Gut–Brain Axis in Adenine-Induced Chronic Kidney Disease Mice
Source: Mar Drugs. 2026 Sep 16;24(9):323. doi: 10.3390/md24090323 (PMC13608249; doi:10.3390/md24090323)
Supplement: Supplementary file 1 [file marinedrugs-24-00323-s001.zip › marinedrugs-4511406-supplementary.pdf]

Supporting Information

Fucus vesiculosus-derived fucoidan mitigates recognition memory deficits in association with modulation of the microbiota-gut-brain axis in adenine-induced chronic kidney disease mice

Xiaobo Kang [1,†], Zhiyou Yang [1,2,†], Zhihui Ma [1], Jinfeng Hong [1], Yi Zhang [1,2], Longjian Zhou [1,2,*]

1 College of Food Science and Technology, Shenzhen Institute of Guangdong Ocean University, Guangdong Provincial Key Laboratory of Aquatic Product Processing and Safety, Guangdong Province Engineering Laboratory for Marine Biological Products, Zhanjiang Municipal Key Laboratory of Marine Drugs and Nutrition for Brain Health, Guangdong Ocean University, Zhanjiang 524088, China; k17701892041@126.com (X.K.); zyyang@gdou.edu.cn (Z.Y.); mazhihui@stu.gdou.edu.cn (Z.M.); jinfeng_hong@163.com (J.H.); zhangyi@gdou.edu.cn (Y.Z.)

2 Collaborative Innovation Center of Seafood Deep Processing, Dalian Polytechnic University, Dalian 116034, China

* Correspondence: zhoulongjian@gdou.edu.cn; Tel.: +86-0759-2396046

† These authors contributed equally to this work.

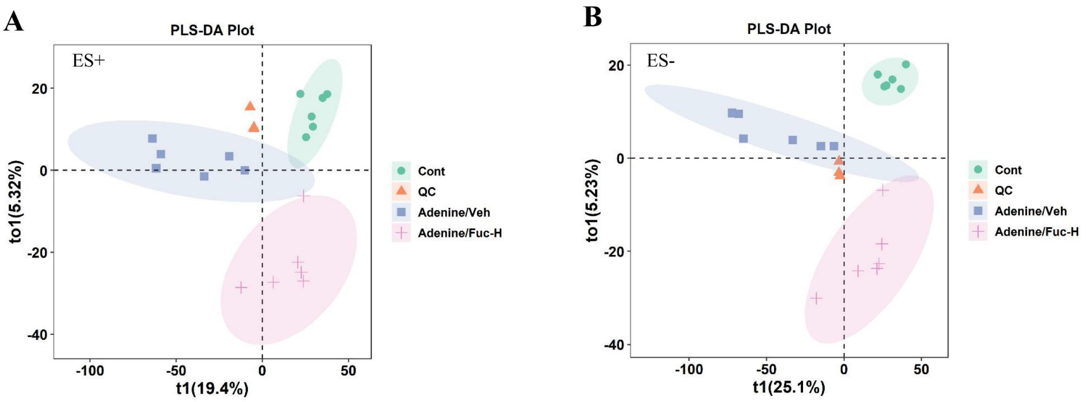

Figure S1. Partial least squares-discriminant analysis (PLS-DA) of serum metabolomic profiles. (A-B) Score plots illustrate the spatial separation of metabolic phenotypes among the experimental groups in positive ionization mode (ES^+) (A) and negative ionization mode (ES^-) (B).

Table S1. The detailed information of reagents used for biochemical analysis.

Name	Catalog No.	Manufacturer	Assay method
IL-10	ZY2176-A	Jiangsu Zeyu Biotechnology Co., Ltd. (Jiangsu, China)	ELISA
IL-4	ZY2165-A	Jiangsu Zeyu Biotechnology Co., Ltd. (Jiangsu, China)	ELISA
IL-1β	ZY2040-A	Jiangsu Zeyu Biotechnology Co., Ltd. (Jiangsu, China)	ELISA
TNF-α	ZY2032-A	Jiangsu Zeyu Biotechnology Co., Ltd. (Jiangsu, China)	ELISA
IFN-γ	MM-0182M1	Jiangsu Meimian Industrial Co., Ltd (Yancheng, China)	ELISA
CAT	A007-1-1	Nanjing Jiancheng Bioengineering Institute (Nanjing, China)	Ammonium molybdate (AM)
MDA	A003-1-2	Nanjing Jiancheng Bioengineering Institute (Nanjing, China)	Thiobarbituric acid (TBA)
SOD	A001-1-1	Nanjing Jiancheng Bioengineering Institute (Nanjing, China)	Hydroxylamine (HA)
GSH-Px	MM-44117M1	Jiangsu Meimian Industrial Co., Ltd (Yancheng, China)	ELISA
ROS	S0039	Beyotime Biotech Inc. (Shanghai, China)	2′,7′-dichlorodihydrofluorescein (DCFH)
